# Neurological Soft Signs and Brain Abnormalities in Schizophrenia: A Literature Review

**DOI:** 10.7759/cureus.11050

**Published:** 2020-10-19

**Authors:** Bindu Rathod, Arveen Kaur, Deepak M Basavanagowda, Devyani Mohan, Nupur Mishra, Sehrish Fuad, Sadia Nosher, Zaid A Alrashid, Stacey E Heindl

**Affiliations:** 1 Psychiatry and Behavioral Sciences, California Institute of Behavioral Neurosciences and Psychology, Fairfield, USA; 2 Surgery, California Institute of Behavioral Neurosciences and Psychology, Fairfield, USA; 3 Medicine, California Institute of Behavioral Neurosciences and Psychology, Fairfield, USA; 4 Internal Medicine, California Institute of Behavioral Neurosciences and Psychology, Fairfield, USA; 5 Family Medicine, California Institute of Behavioral Neurosciences and Psychology, Fairfield, USA; 6 Neurology, California Institute of Behavioral Neurosciences and Psychology, Fairfield, USA; 7 Medicine, Avalon University School of Medicine, Willemstad, CUW

**Keywords:** neurological soft signs, schizophrenia, nss, brain changes, magnetic resonance imaging, voxel-based morphometry

## Abstract

Neurological soft signs (NSS) are subtle neurological impairments in sensory integration, motor coordination, balance, and sequencing of complex motor acts. The prevalence of NSS is well over 50% in schizophrenic patients compared to about 5% in healthy controls. About 30% of schizophrenia patients are resistant to treatment. The main reason for not finding better pharmaceutical agents is the inability to elicit the underlying neurophysiological and neuroanatomical basis of schizophrenia. The most common NSS can be divided into three domains: motor coordination, sequencing of complex motor acts, and sensory integration. Here, the neuroimaging correlates of the abovementioned NSS are reviewed. Most of the studies found a negative correlation of NSS subs cores motor coordination and complex motor tasks with the cerebellum, inferior frontal gyrus, and postcentral gyrus. There was a negative correlation between cortical thickness and NSS total scores in the left paracentral lobule, precuneus, middle frontal cortex, right inferior temporal cortex, left/right superior parietal cortex. Instead of considering NSS as a mere trait or state markers, its active inclusion in patient management is required to improve patients' quality of life. Future studies on larger cohorts, combining different imaging modalities are needed to elucidate how these factors might relate to each other and contribute to NSS.

## Introduction and background

Neurological soft signs (NSS) are defined as subtle neurological impairments in sensory integration, motor coordination, balance, and sequencing of complex motor acts [1]. The name "soft signs" indicate that they have poor localizing value. Although NSS are found in many neuropsychiatric conditions, they have a much higher prevalence in schizophrenia [2]. When considering all patients with schizophrenia, that is, first episode unmedicated, medicated patients, and those with chronic schizophrenia, the overwhelming majority of studies report a prevalence of well over 50% with abnormal NSS, compared to about 5% in normal healthy controls [3].

The amount of NSS and the single sign's abnormality may even differentiate between sporadic and familial schizophrenia with more striking abnormalities in familial cases [4]. Higher NSS scores are seen in patients compared to healthy controls and in the patient's relatives, the score is positively correlated with the degree of genetic relationship [5].

Some of the instruments developed for the measurement of NSS are the neurological soft signs scale [6], the neurological evaluation scale (NES) [7], the quantified neurological scale [8], Heidelberg scale [1], Cambridge neurological inventory [9], the condensed neurological examination [10] and brief motor scale [11].

NSS in schizophrenia indicates both trait (i.e., genetic liability) and state (i.e., acuity of the disease process) related factors [12]. NSS tends to decrease in patients who show adequate response to treatment, whereas persist or increase in patients with inadequate response to treatment [13]. NSS correlates with negative symptoms and cognitive dysfunction in patients with schizophrenia. NSS is much more common in childhood-onset schizophrenia and adolescent-onset schizophrenia compared to adult-onset schizophrenia patients. NSS are markers of neurodevelopmental abnormalities in schizophrenia as evidenced by non-suppression of primitive reflexes with cortical maturation [14].

Childhood-onset schizophrenia (COS) is associated with negative symptoms, cognitive dysfunction, and inadequate response to treatment. Childhood-onset psychosis can exert a disruptive effect on the essential functions of personality and in youth, appears to be associated with severe socioeconomic consequences in the further course of the disorder [15]. Long-term longitudinal studies are lacking in patients with COS regarding NSS and morphological changes in the brain. Although COS is a rare disorder, its study can provide a better understanding of the neurological basis of differences between COS and adult schizophrenia.

Treatment-resistant schizophrenia (TRS) affects approximately 30% of patients with schizophrenia [16]. The major reason behind not finding better pharmaceutical agents is the inability to elicit the underlying neurophysiological and neuroanatomical basis of schizophrenia. With the advancement of neurological imaging studies, it is possible to monitor morphological changes in the brain of patients with schizophrenia. If we can correlate NSS with structural changes in the brain, we can decipher neuropathological pathways of schizophrenia. This offers a hope to develop newer agents to treat schizophrenia, predict schizophrenia in children born with neurodevelopmental deficits, and use NSS as prognostic markers of schizophrenia.

The present study aims to fill the gaps in the current knowledge base regarding the importance of correlating NSS with the morphological changes in the brain of patients with schizophrenia. With a better understanding of underlying neurological pathways, we can offer a more comprehensive management plan to the patients with schizophrenia.

## Review

**Figure 1:**
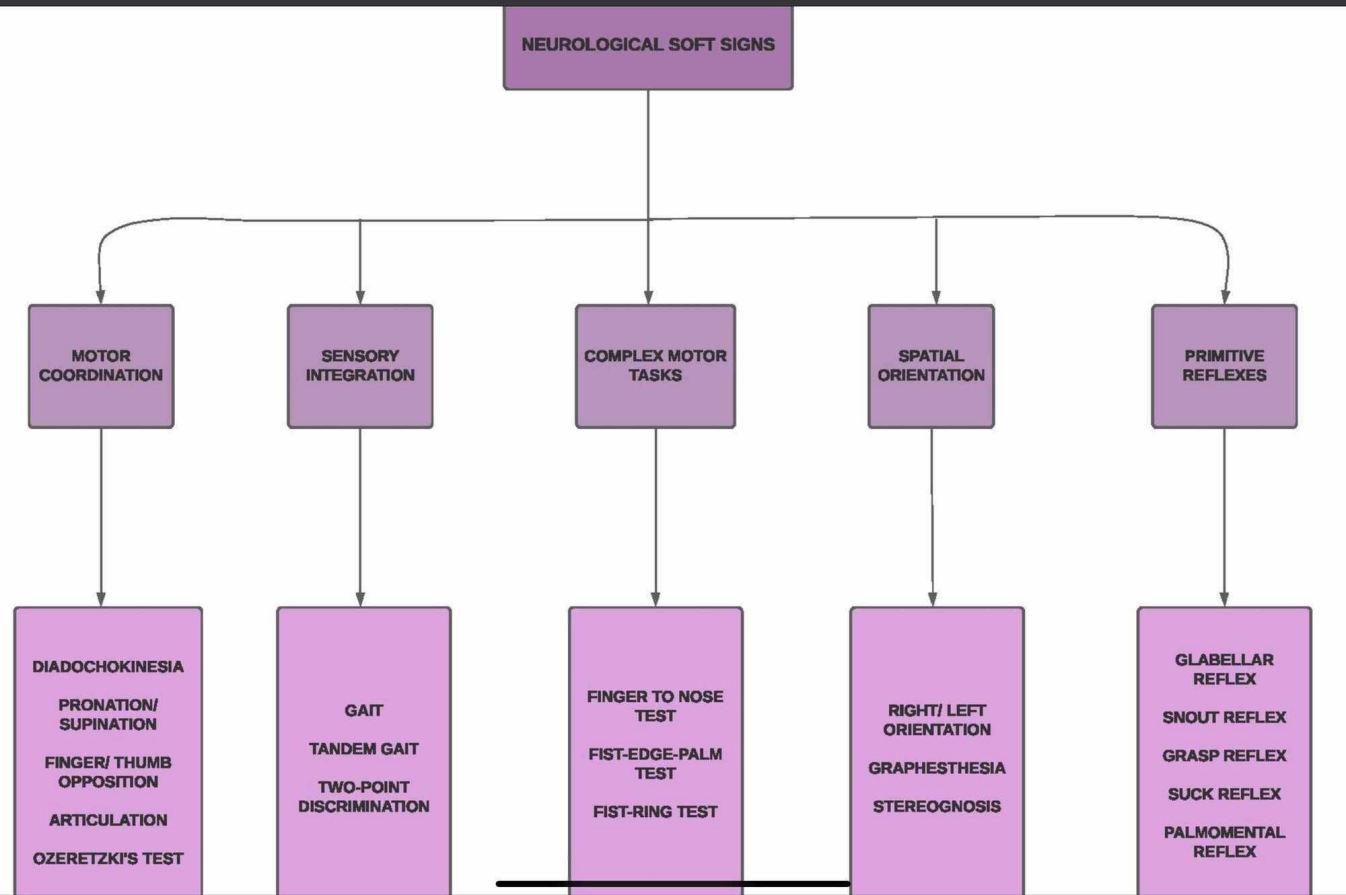
Neurological soft signs in patients with schizophrenia

NSS are present in patients with schizophrenic patients irrespective of the use of neuroleptic drugs [17]. The most commonly found NSS can be divided into three domains: motor coordination, sequencing of complex motor acts, and sensory integration [18]. Difficulties in motor coordination include intention tremor, balance and gait abnormalities, finger-thumb opposition, dysdiadochokinesis, and general coordination. The sequencing of complex motor acts is assessed using the fist-edge-palm test, fist-ring test, and the Ozeretski test. Impaired sensory integration is manifested as bilateral extinction, impaired audiovisual integration, agraphesthesia, astereognosis, and right-left confusion. Hard signs are assessed using arm holding test and mirror movements. Listed in Figure 1 are the various NSS seen in patients with schizophrenia.

Correlation of NSS with motor and sensory dysfunction

The main findings of a study indicated a lateralization effect in healthy controls with a greater left than right hemispheric sensorimotor cortex coactivation during ipsilateral finger-to-thumb opposition. Compared with the healthy controls, the schizophrenic patients showed a decreased activation of both sensorimotor cortices and supplementary motor area (SMA) and a reversed lateralization effect. This may be an indication that the sensorimotor cortex and SMA dysfunction may contribute to NSS. However, this study could not explain the reason behind the reversed lateralization pattern [19].

Another study showed that patients with schizophrenia had a diminished activation of the sensorimotor cortices at an increased variability of motor performance but did not differ significantly from healthy controls with respect to the frequency and amplitude of the pronation/supination movements. The limited temporal and spatial resolution capacity of the functional Magnetic Resonance Imaging (fMRI) technique used was a potential confounding variable. Also, some cerebral sites necessary for motor functioning, particularly the basal ganglia and the cerebellum, were not investigated [20].

The vital role of the cerebellum in motor coordination is well-founded. These functions are known to be lacking in patients with schizophrenia and probands with increased genetic liability [21]. Clinically these deficits are present as NSS. In Keshavan et al.’s study, cognitive/perceptual neurological examination abnormalities in schizophrenic patients were significantly and strongly correlated with smaller volumes in the left heteromodal association cortex and the cerebellum and were less strongly correlated with caudate volumes. On the other hand, motor abnormalities correlated with smaller right and left caudate and cerebellar volumes but not with heteromodal association cortex volumes. Interviewer bias was the primary limitation of this study since examiners were not blind to subjects' symptoms and histories. There was also significant intersubject variability in the anatomical landmarks, which made the heteromodal association cortex's precise partition difficult [22].

A study of first-episode patients with schizophrenia found decreased cerebellar volumes bilaterally compared to healthy controls. The study also found that decreased volumes of the right cerebellar hemisphere in patients were associated with elevated NSS scores. The study's main limitations were a failure to delineate cerebellar subdivisions and determine whether these are selectively affected in schizophrenia or a general deficit to the cerebellum [23].

A study showed a negative correlation between motor integration score and grey matter (GM) volume in the cerebellum, right inferior frontal gyrus, left postcentral gyrus, and right occipital gyrus. Besides, sensory integration scores were negatively correlated with GM volume in clusters located in the right and left cerebellum, even after correcting for age and sex. White matter analysis showed a negative correlation between motor coordination score and left frontal medial gyrus volume and a positive correlation with right precuneus volume. High-NSS patients had reduced GM volume in the left postcentral gyrus and reduced white matter volume in the right insular region and left cerebellar lobe. The study was limited by the small sample size [24].

In a study, logistic regression analysis showed that only the Motor Sequencing Signs (MSS) sub-score was a significant predictor of the subject's status among the NSS sub-scores. Optimized voxel-based morphometry (VBM) analysis showed that the MSS sub-score had a significant negative correlation with total and regional GM volumes (prefrontal, posterior cingulate, temporal cortices, putamen, and cerebellum) in schizophrenia patients but not in controls. This study was limited by small sample size, an imbalance in the sex ratio, and relatively late age of onset in the patient group [25].

In Thomann et al.’s study, NSS scores were significantly higher in schizophrenic patients than in controls. The cerebella of patients were significantly smaller with atrophy pronounced in the corpus medullare bilaterally. Higher NSS scores in the patients' group were associated with reduced volumes of the cerebellum's posterior lobes. In contrast, no significant associations between NSS scores and cerebellar sub-regions in healthy subjects emerged [26].

In another study done by the same first author, increased scores on the subscale right/left and spatial orientation was correlated with GM loss in the cerebellar vermis. Higher NSS levels were also associated with a reduced white-matter density in the right inferior frontal gyrus (NSS total score), the left inferior frontal gyrus (NSS total score, motor coordination, complex motor tasks), the right cerebellum (NSS total score, motor coordination) and the corpus callosum (motor coordination) [27].

A recent study showed a progressive cognitive decline in patients with chronic schizophrenia along with increases on the NSS subscales of "motor coordination" and "integrative functions." However, the study was limited by confounding factors of diabetes mellitus, metabolic syndrome, small sample size, and patients' inability to come for follow-up [28].

Table 1 summarizes studies showing the correlation of NSS with motor and sensory dysfunction.

**Table 1:** Studies showing correlation of NSS with motor and sensory dysfunction NSS: neurological soft signs, SMA: supplementary motor area, MSS: Motor Sequencing Signs

Study	Year	Sample Size	Conclusion
Schröder et al. [19]	1995	Cases-10 Controls- 7	Sensorimotor cortex and SMA dysfunction are associated with motor disturbances in schizophrenia.
Schröder et al. [20]	1999	Cases-12 Controls- 12	A decreased sensorimotor cortex and SMA activation in schizophrenia and suggests that these changes may correspond to an increased motor variability.
Keshavan et al. [22]	2003	Cases- 90 Controls- 93	Neurological abnormalities may reflect discrete neuroanatomical changes in schizophrenia and may have a localizing value.
Bottler et al. [23]	2005	Cases- 37 Controls- 18	Cerebellar changes in schizophrenia are associated with NSS.
Mouchet-Mages et al. [24]	2007	Cases- 21 Controls- 0	Non-motor dysfunction is associated with cerebellar structural changes in schizophrenia.
Venkatasubramanian et al. [25]	2008	Cases- 30 Controls- 27	Cortical and cerebellar correlates of the sub-score MSS support the concept of "cognitive dysmetria " in schizophrenia.
Thomann et al. [26]	2009	Cases- 30 Controls- 21	Alterations in distinct cerebellar regions are related to NSS in schizophrenia
Thomann et al. [27]	2009	Cases- 42 Controls- 22	The pattern of cerebral changes associated with NSS supports the model of ‘cognitive dysmetria’ with a disrupted cortico-cerebellar-thalamic-cortical circuit in schizophrenia.
Herold et al. [28]	2020	Cases- 21 Controls- 0	Executive dysfunctions may occur due to premature brain aging in schizophrenia.

Correlation of NSS with different lobes of the brain

In a study using computed tomography (CT) and positron emission tomography (PET), the delusional subsyndrome was associated with a decreased hippocampal function, whereas the negative subsyndrome exhibited prominent hypofrontality and left temporal cortex changes. The delusional and the negative subsyndrome were associated with a decreased activity in the medial frontal gyrus compared to the other schizophrenic patients and the healthy controls. The disorganized subsyndrome was characterized by overactivity in the parietal cortex and motor strip and reduced corpus callosum activity. This was the only study done to categorize schizophrenia into different subsyndromes based on neurological substrates [29].

Lateral ventricles enlargement was positively correlated with the right stereo agnosia item in Bersani et al.’s study done on chronic schizophrenic patients using MRI. Positive correlations were shown between the third ventricular width and the index finger-right-thumb opposition, the index finger-left-thumb opposition, and the Sensorial Integration area's total score. Medial temporal lobe atrophy correlated with the right stereo agnosia item, with the index finger-right-thumb opposition and with the index finger-left-thumb opposition, as well as with the face-hand extinction. Finally, cerebellar vermis atrophy was positively correlated with the rhythmic drumming item, the index finger-right-thumb opposition, the index finger-left-thumb opposition, the specular motions at index finger-left-thumb opposition, the face-hand extinction, the right-left confusion, and with the left index finger-nose test. The main limitations of the study were the small sample size and no healthy controls [30].

A study was conducted to compare NSS in patients of schizophrenia with onset in childhood (COS), adolescence, and adulthood. COS patients showed maximum dysfunction in the frontotemporal lobes and to a lesser extent in the parietal lobe. There was an anatomical gradient in COS patients with more severe frontal lobe involvement followed by temporal and then parietal lobe. NSS correlated negatively with IQ, educational level, and age of onset, and positively with psychopathology scores. This study was limited by the absence of healthy controls, small sample size, and inclusion of patients on neuroleptic medication. Cerebral dominance was not tested due to the Cox and Ludwig scale's limitations in the study. This was the only study done on COS regarding NSS [18].

In a study using optimized VBM, higher NSS scores in the patient group were associated with a decreased GM density of the thalamus bilaterally, the left post-central gyrus, the right lingual gyrus, the left insula, the right precentral gyrus, the head of the right caudate nucleus, and the left hemisphere of the cerebellum [27].

Higher NSS total scores were associated with bilateral volume alterations of the thalamus, caudate nucleus, putamen, and atrophy of the right pallidum in a study done on schizophrenia patients using high-resolution MRI at 3 Tesla(T). Similar associations were found with motor coordination scores. Higher scores on subscale complex motor tasks were associated with the left caudate nucleus volume changes, whereas those on subscale orientation were negatively correlated with the right thalamus and right pallidum volume. In contrast, there were no significant associations between volumetric measures and scores on the subscale hard signs and integrative functions. The vertex-wise analysis found higher NSS total scores were associated with anterior shrinkage of the right thalamus and with left caudate nucleus atrophy mainly affecting anterior, posterior, and medial part. Furthermore, elevated NSS total scores were associated with shape alterations of the right-hemispheric internal pallidum. Increased scores on subscale motor coordination were accompanied by bi-hemispheric thalamic shrinkage, mainly affecting its right anterior part and medial atrophy of the right pallidum. The study's significant limitations were modest sample size, the use of antipsychotic medications, and the cross-sectional design [31].

Longitudinal GM changes were measured using tensor-based morphometry (TBM) in Kong et al.’s study performed on patients with first-episode schizophrenia. At follow-up after one year, patients showed significantly reduced NSS scores. For further analysis, the patient sample was dichotomized into patients with declining NSS scores and patients with persistently elevated scores, respectively. While patients with decreasing NSS displayed only localized change within the left frontal lobe, cerebellum, and cingulate gyrus, patients with persistently increased scores showed marked GM reductions of the sub-lobar claustrum, right cingulate gyrus, right cerebellum, left frontal lobe, and left middle frontal gyrus. The study was limited by the small sample size [32].

Higher NSS scores and higher scores on subscales motor coordination, complex motor tasks, and hard signs were significantly associated with brainstem volume reduction in a study done on first-episode schizophrenia patients. In contrast, no significant associations between volumetric measures and scores on the subscale right/left and spatial orientation and integrative functions were found. According to vertex-wise shape analysis, these correlations referred to regionally specific morphometric alterations, mostly in the pons and midbrain, rather than to the brainstem's global atrophy. The study was limited by the modest sample size, treatment with antipsychotic medications, and the cross-sectional design. This was the only study done on the correlation of NSS with brainstem morphology [33].

A recent study showed that the patient group's NSS total scores were significantly correlated to reduced GM in the right lingual gyrus, left parahippocampal gyrus, left superior temporal gyrus, left thalamus (medial dorsal nucleus), and left posterior lobe of the cerebellum (declive). Respective negative associations could also be revealed for the subscales "motor coordination," "complex motor tasks" and "right/left and spatial orientation." The examiners' bias limited the study as they were aware of the patient group [34].

Table 2 summarizes studies showing the correlation of NSS with different lobes of the brain. 

**Table 2:** Studies showing correlation of NSS with different lobes of brain NSS: neurological soft signs

Study	Year	Sample Size	Conclusion
Schröder et al. [29]	1995	CT Scan: Cases- 50 PET Scan: Cases- 79 Controls- 47	The subsyndromes of schizophrenia appear to be characterized by deviant patterns of cerebral alterations, rather than deficits in a single location.
Bersani et al. [30]	2007	Cases- 33 Controls- 0	There is a correlation between NSS and neuroanatomical alterations in schizophrenia.
Biswas et al. [14]	2007	Cases- 55 Controls- 0	Childhood onset schizophrenia may be more strongly associated with a generalized disruption of brain function.
Thomann et al. [27]	2009	Cases- 42 Controls- 22	The pattern of cerebral changes associated with NSS supports the model of ‘cognitive dysmetria’ with a disrupted cortico-cerebellar-thalamic-cortical circuit in schizophrenia.
Hirjak et al. [31]	2012	Cases- 20 Controls- 0	NSS is associated with morphometric alterations of multiple subcortical regions in schizophrenia.
Kong et al. [32]	2012	Cases- 20 Controls- 20	NSS may help establish prognosis in first-episode patients with schizophrenia.
Hirjak et al. [33]	2013	Cases- 21 Controls- 0	The brainstem is involved in the pathogenesis and severity of NSS.
Herold et al. [34]	2020	Cases- 49 Controls- 29	Progressive cerebral changes are associated with persistent NSS scores in patients with an adverse course of the disorder.

Correlation of NSS with cortical morphology

In Gay et al.’s study on cortical morphology and NSS, schizophrenia patients with NSS showed significant overall cortical sulcation reductions in both hemispheres compared to non-significant NSS patients. Exploratory regional analyses also revealed correlations of the motor coordination subscore with the regional sulcation of the left dorsolateral prefrontal cortex (DLPFC). The sensory integration subscore correlated with the right lateral parietal cortex, including the associative Brodmann areas five and seven. Additionally, the motor integration subscore correlated with the sulcation of the left medial parieto-occipital cortex. The study's main limitations were the small sample size and the absence of healthy controls [35].

Cortical thickness refers to a measure of GM morphology in cortical regions. GM's morphological changes proceed progressively from subcortical to cortical regions during the course of schizophrenia [36]. Cortical thickness is calculated as the closest distance from the gray/white boundary to the gray/cerebrospinal fluid boundary at each vertex on the tessellated surface on MRI [37].

A study done on recent-onset schizophrenia patients using high-resolution MRI of whole-brain found negative correlations between cortical thickness and higher NSS total scores in the left paracentral lobule and precuneus. Positive correlations between cortical thickness and higher NSS total scores were found in the middle temporal gyrus bilaterally. Negative correlations between cortical thickness and higher NSS scores on the subscales "hard signs" were detected in the left postcentral gyrus. Positive correlations between cortical thickness and higher NSS scores on the subscale "complex motor tasks" were found in the middle temporal gyrus bilaterally. Additionally, a positive correlation was identified between cortical thickness in the left postcentral gyrus and higher NSS scores on the subscale "left/right and spatial orientation." Higher NSS scores on the subscale "integrative functions" were positively correlated to cortical thickness in the left middle temporal and supramarginal gyrus. The study also detected negative correlations between higher NSS scores on the subscale "integrative functions" and cortical thickness in the right paracentral lobule, inferior parietal lobe, and precuneus. These findings suggest that cortical thickness may serve as a very sensitive metric and as a possible indicator of a distinct process in schizophrenia patients with NSS. The study's main limitations were a cross-sectional design, a relatively modest sample size, and no healthy controls [38].

In a study done by the same author on the local gyrification index (LGI) among patients with recent-onset schizophrenia, higher sensory and spatial NSS were negatively associated with LGI changes in the left precentral gyrus and the left precuneus. Additionally, higher motor NSS were positively related to LGI changes, mainly in the supramarginal gyrus bilaterally, the right superior parietal area, and the left superior temporal gyrus. LGI refers to the extent of cortical folding majority of which occurs during the prenatal period [39]. This study was limited by small sample size, a cross-sectional design, the use of antipsychotic medications, and no control group [40].

In another study, patients with chronic schizophrenia had significantly increased NSS compared to controls. NSS inpatients had a significant negative correlation with cortical thickness in the left pars triangularis, left rostral middle frontal cortex, right inferior temporal cortex, left/right superior parietal cortex, right postcentral cortex, and right supramarginal cortex. These areas are essential for motor and sensory functions. In contrast, a significant negative correlation in healthy controls prevailed in the left rostral anterior cingulate, pericalcarine, and right superior/middle temporal cortices. This showed that patients and controls have different anatomical substrates of NSS. This study was limited by small sample size [41].

Table 3 summarizes studies showing the correlation of NSS with cortical morphology. 

**Table 3:** Studies showing correlation of NSS with cortical morphology NSS: neurological soft signs

Study	Year	Sample Size	Conclusion
Gay et al. [35]	2013	Cases- 44 Controls- 0	There are distinct neurodevelopmental pathways in patients with NSS compared to non-significant NSS patients.
Hirjak et al. [38]	2014	Cases- 28 Controls- 0	There is a significant relationship between cortical thickness and the extent of NSS in schizophrenia.
Hirjak et al. [40]	2015	Cases- 33 Controls- 0	Cortical folding patterns may play an important role in the development of NSS in schizophrenia.
Kong et al. [41]	2015	Cases- 18 Controls- 20	Patients and controls have different anatomical substrates of NSS.

Correlation of NSS with faulty brain network

In a study, patients with "high" scores on the complex motor act sequencing subscale showed a smaller corpus callosum (CC) rostral body, while patients with "high" scores on the integrative sensory function subscale showed a smaller CC splenium. For both NES total and complex motor acts sequencing subscale, an increase of the CC genu accompanied "high" scores. Correlational analyses revealed a significant inverse relationship between the CC rostral body size and the sequencing of complex motor acts subscale score. A significant positive correlation was shown between CC genu size and both NES total and complex motor act sequencing subscale scores. An association between NSS and CC morphology may indicate a disturbed functional interhemispheric connectivity in schizophrenic patients. The study was limited by the absence of health controls [42].

Using diffusion tensor magnetic resonance images (DT-MRI) and tractography, a study showed impaired integrity of the left superior cerebellar peduncle (SCP) in schizophrenia patients with deficits in the sequencing of motor acts. This is the only study done to explore the white matter tracts associated with NSS. The study's significant limitations were small sample size, a study of a selected part of the network, and the use of antipsychotic medicines [43].

A cross-sectional study examined functional connectivity abnormalities of the default mode network (DMN) associated with NSS in schizophrenia. Abnormal connectivity of the caudate core with other areas that make up the DMN may underpin the functional and structural abnormalities associated with NSS. NSS scores differed significantly between groups, ranging from higher to lower scores for patients, unaffected relatives, and healthy controls. The connectivity analysis revealed significant hyperconnectivity in the fusiform gyrus, insular and dorsolateral prefrontal cortices, inferior and middle frontal gyri, middle and superior temporal gyri, and posterior cingulate cortex in patients with schizophrenia as compared to controls. Also, unaffected relatives showed hyperconnectivity in relation to controls in the supramarginal association and dorsal posterior cingulate cortices in patients with schizophrenia compared to in controls. Additionally, unaffected relatives exhibited hyperconnectivity relative to controls in the supramarginal association and dorsal posterior cingulate cortices and the anterior prefrontal cortex. A negative correlation was found between left caudate connectivity and NSS. The study was limited by small sample size and examiners' bias [44].

Table 4 summarizes studies showing the correlation of NSS with a faulty brain network. 

**Table 4:** Studies showing correlation of NSS with faulty brain network NSS: neurological soft signs, CC: corpus callosum, DMN: default mode network

Study	Year	Sample Size	Conclusion
Bersani et al. [42]	2011	Cases- 29 Controls- 0	An association between NSS and CC morphology may indicate a disturbed functional connectivity in schizophrenic patients.
Hüttlova et al. [43]	2014	Cases- 24 Controls- 23	Schizophrenia patients with deficits in sequencing complex motor acts showed impaired integrity of the left superior cerebellar peduncle
Galindo et al. [44]	2018	Cases- 27 Unaffected relatives-23 Controls- 35	Widespread abnormal connectivity in schizophrenia, strengthening DMN hyperconnectivity and NSS as neurobiological markers of schizophrenia.

Limitations

This paper's main limitation was that there were very few studies performed on NSS and brain abnormalities in schizophrenia that we could base our review on. We did not undertake a systematic review in our study and no quality assessment of the selected research studies was carried out. Moreover, inconsistencies between the selected studies could be partially explained by the MRI data acquisition at various field strengths, i.e., 1.5 T versus 3 T in more recent studies and different methods of morphometric measurements used in various studies.

## Conclusions

In summary, most studies showed higher NSS scores in schizophrenia patients than healthy controls. Most of the studies found a negative correlation of NSS subscores motor coordination and complex motor tasks with the cerebellum, inferior frontal gyrus, and postcentral gyrus. There was a negative correlation between cortical thickness and NSS total scores in the left paracentral lobule, precuneus middle frontal cortex, right inferior temporal cortex, left/right superior parietal cortex.

Rather than viewing NSS as a mere trait or state markers, its active inclusion in patient management is needed to improve patients' quality of life. Even though the exact nature of underlying biological processes and potential interaction between different brain regions remains elusive, we must continue to search for better research methodologies to understand the neurobiological basis of schizophrenia. Future studies on larger cohorts, combining different imaging modalities including volumetry, diffusion tensor, and functional imaging are needed to elucidate how these factors might relate to each other and contribute to NSS.

## References

[1] Schroder J, Niethammer R, Geider FJ, Reitz C, Binkert M, Jauss M, Sauer H (1991). Neurological soft signs in schizophrenia. Schizophr Res.

[2] Heinrichs DW, Buchanan RW (1988). Significance and meaning of neurological signs in schizophrenia. Am J Psychiatry.

[3] Chan RC, Chen EY (2007). Neurological abnormalities in Chinese schizophrenic patients. Behav Neurol.

[4] Scheffer RE (2004). Abnormal neurological signs at the onset of psychosis. Schizophr Res.

[5] Cantor-Graae E, McNeil TF, Rickler KC, Sjostrom K, Rawlings R, Higgins ES, Hyde TM (1994). Are neurological abnormalities in well discordant monozygotic cotwins of schizophrenic subjects the result of perinatal trauma?. Am J Psychiatry.

[6] Quitkin F, Rifkin A, Klein DF (1976). Neurologic soft signs in schizophrenia and character disorders: organicity in schizophrenia with premorbid asociality and emotionally unstable character disorders. Arch Gen Psychiatry.

[7] Buchanan RW, Heinrichs DW (1989). The neurological evaluation scale (NES): a structured instrument for the assessment of neurological signs in schizophrenia. Psychiatry Res.

[8] Convit A, Volavka J, Czobor P, de Asis J, Evangelista C (1994). Effect of subtle neurological dysfunction on response to haloperidol treatment in schizophrenia. Am J Psychiatry.

[9] Chen EYH, Shapleske J, Luque R (1995). The Cambridge neurological inventory: a clinical instrument for assessment of soft neurological signs in psychiatric patients. Psychiatry Res.

[10] Rossi A, De Cataldo S, Di Michele V, Manna V, Ceccoli S, Stratta P, Casacchia M (1990). Neurological soft signs in schizophrenia. Br J Psychiatry.

[11] Jahn T, Cohen R, Hubmann W (2006). The brief motor scale (BMS) for the assessment of motor soft signs in schizophrenic psychoses and other psychiatric disorders. Psychiatry Res.

[12] Bachmann S, Schröder J (2018). Neurological soft signs in schizophrenia: an update on the state- versus trait-perspective. Front Psychiatry.

[13] Bachmann S, Degen C, Geider FJ, Schröder J (2014). Neurological soft signs in the clinical course of schizophrenia: results of a meta-analysis. Front Psychiatry.

[14] Biswas P, Malhotra S, Malhotra A, Gupta N (2007). Comparative study of neurological soft signs in schizophrenia with onset in childhood, adolescence and adulthood. Acta Psychiatr Scand.

[15] Eggers C, Bunk D (1997). The long-term course of childhood-onset schizophrenia: a 42-year followup. Schizophr Bull.

[16] Meltzer HY (1997). Treatment-resistant schizophrenia - the role of clozapine. Curr Med Res Opin.

[17] Bombin I, Arango C, Buchanan RW (2005). Significance and meaning of neurological signs in schizophrenia: two decades later. Schizophr Bull.

[18] Biswas P, Malhotra S, Malhotra A, Gupta N (2007). Comparative study of neurological soft signs in schizophrenia with onset in childhood, adolescence and adulthood. Acta Psychiatr Scand.

[19] Schroeder J, Wenz F, Schad LR, Baudendistel K, Knopp MV (1995). Sensorimotor cortex and supplementary motor area changes in schizophrenia. A study with functional magnetic resonance imaging. Br J Psychiatry.

[20] Schröder J, Essig M, Baudendistel K (1999). Motor dysfunction and sensorimotor cortex activation changes in schizophrenia: a study with functional magnetic resonance imaging. Neuroimage.

[21] Niethammer R, Weisbrod M, Schiesser S (2000). Genetic influence on laterality in schizophrenia? A twin study of neurological soft signs. Am J Psychiatry.

[22] Keshavan MS, Sanders RD, Sweeney JA, Diwadkar VA, Goldstein G, Pettegrew JW, Schooler NR (2003). Diagnostic specificity and neuroanatomical validity of neurological abnormalities in first-episode psychoses. Am J Psychiatry.

[23] Bottmer C, Bachmann S, Pantel J (2005). Reduced cerebellar volume and neurological soft signs in first-episode schizophrenia. Psychiatry Res Neuroimaging.

[24] Mouchet-Mages S, Cachia A, Canceil O (2007). Sensory dysfunction is correlated to cerebellar volume reduction in early schizophrenia. Schizophr Res.

[25] Venkatasubramanian G, Jayakumar PN, Gangadhar BN, Keshavan MS (2008). Neuroanatomical correlates of neurological soft signs in antipsychotic-naive schizophrenia. Psychiatry Res Neuroimaging.

[26] Thomann PA, Roebel M, Santos VD, Bachmann S, Essig M, Schröder J (2009). Cerebellar substructures and neurological soft signs in first-episode schizophrenia. Psychiatry Res Neuroimaging.

[27] Thomann PA, Wüstenberg T, Santos VD, Bachmann S, Essig M, Schröder J (2009). Neurological soft signs and brain morphology in first-episode schizophrenia. Psychol Med.

[28] Herold CJ, Duval CZ, Schröder J (2020). Neurological soft signs and cognition in the late course of chronic schizophrenia: a longitudinal study. Eur Arch Psychiatry Clin Neurosci.

[29] Schröder J, Buchsbaum MS, Siegel BV, Geider FJ, Niethammer R (1995). Structural and functional correlates of subsyndromes in chronic schizophrenia. Psychopathology.

[30] Bersani G, Paolemili M, Quartini A (2007). Neurological soft signs and cerebral measurements investigated by means of MRI in schizophrenic patients. Neurosci Lett.

[31] Hirjak D, Wolf RC, Stieltjes B, Seidl U, Schröder J, Thomann PA (2012). Neurological soft signs and subcortical brain morphology in recent onset schizophrenia. J Psychiatr Res.

[32] Kong L, Bachmann S, Thomann PA, Essig M, Schröder J (2012). Neurological soft signs and gray matter changes: a longitudinal analysis in first-episode schizophrenia. Schizophr Res.

[33] Hirjak D, Wolf RC, Stieltjes B (2013). Neurological soft signs and brainstem morphology in first-episode schizophrenia. Neuropsychobiology.

[34] Herold CJ, Essig M, Schröder J (2020). Neurological soft signs (NSS) and brain morphology in patients with chronic schizophrenia and healthy controls. PLoS One.

[35] Gay O, Plaze M, Oppenheim C (2012). Cortex morphology in first-episode psychosis patients with neurological soft signs. Schizophr Bull.

[36] Ellison-Wright I, Glahn DC, Laird AR, Thelen SM, Bullmore E (2008). The anatomy of first-episode and chronic schizophrenia: an anatomical likelihood estimation meta-analysis. Am J Psychiatry.

[37] Fischl B, Dale AM (2000). Measuring the thickness of the human cerebral cortex from magnetic resonance images. Proc Natl Acad Sci USA.

[38] Hirjak D, Wolf RC, Stieltjes B, Hauser T, Seidl U, Schröder J, Thomann PA (2013). Cortical signature of neurological soft signs in recent onset schizophrenia. Brain Topogr.

[39] Palaniyappan L, Park B, Balain V, Dangi R, Liddle P (2014). Abnormalities in structural covariance of cortical gyrification in schizophrenia. Brain Struct Funct.

[40] Hirjak D, Kubera KM, Wolf RC, Thomann AK, Hell SK, Seidl U, Thomann PA (2015). Local brain gyrification as a marker of neurological soft signs in schizophrenia. Behav Brain Res.

[41] Kong L, Herold CJ, Lässer MM (2015). Association of cortical thickness and neurological soft signs in patients with chronic schizophrenia and healthy controls. Neuropsychobiology.

[42] Bersani G, Quartini A, Paolemili M, Clemente R, Iannitelli A, Di Biasi C, Gualdi G (2011). Neurological soft signs and corpus callosum morphology in schizophrenia. Neurosci Lett.

[43] Hüttlova J, Kikinis Z, Kerkovsky M (2014). Abnormalities in myelination of the superior cerebellar peduncle in patients with schizophrenia and deficits in movement sequencing. Cerebellum.

[44] Galindo L, Bergé D, Murray GK, Mané A, Bulbena A, Pérez V, Vilarroya O (2018). Default mode network aberrant connectivity associated with neurological soft signs in schizophrenia patients and unaffected relatives. Front Psychiatry.

